# A systematic review and guide for using multi-response statistical models in co-infection research

**DOI:** 10.1098/rsos.231589

**Published:** 2024-10-04

**Authors:** Francisca Powell-Romero, Konstans Wells, Nicholas J. Clark

**Affiliations:** ^1^ School of Veterinary Science, The University of Queensland, 5391 Warrego Hwy, Gatton, Queensland 4343, Australia; ^2^ Department of Biosciences, Swansea University, Singleton Park, Swansea SA2 8PP, UK

**Keywords:** co-infection, ecology, epidemiology, multi-response, multivariate, statistical modelling

## Abstract

The simultaneous infection of organisms with two or more co-occurring pathogens, otherwise known as co-infections, concomitant infections or multiple infections, plays a significant role in the dynamics and consequences of infectious diseases in both humans and animals. To understand co-infections, ecologists and epidemiologists rely on models capable of accommodating multiple response variables. However, given the diversity of available approaches, choosing a model that is suitable for drawing meaningful conclusions from observational data is not a straightforward task. To provide clearer guidance for statistical model use in co-infection research, we conducted a systematic review to (i) understand the breadth of study goals and host–pathogen systems being pursued with multi-response models and (ii) determine the degree of crossover of knowledge among disciplines. In total, we identified 69 peer-reviewed primary studies that jointly measured infection patterns with two or more pathogens of humans or animals in natural environments. We found stark divisions in research objectives and methods among different disciplines, suggesting that cross-disciplinary insights into co-infection patterns and processes for different human and animal contexts are currently limited. Citation network analysis also revealed limited knowledge exchange between ecology and epidemiology. These findings collectively highlight the need for greater interdisciplinary collaboration for improving disease management.

## 1. Introduction

Simultaneous infection with multiple pathogens, or co-infections, holds significant importance in infectious disease research. The possible detrimental effects of co-infection on host health have been reported for many pathogens, including those from secondary bacterial infections during the 1918 influenza and the Coronavirus disease 2019 (COVID-19) pandemics [1,2]. Other studies have highlighted their significance by observing high frequencies of pathogen co-occurrence in both animals [3,4] and humans [5,6], suggesting that co-infections may be much more common than anticipated in epidemiological research and prevention programmes. As recognition of this has increased in recent years, so too has the availability of multi-pathogen occurrence data, offering researchers vast opportunities to characterize co-infections, provide new insights into disease dynamics and better explain pathogen co-occurrence, which are essential to improving surveillance and disease control.

At the host level, co-infection risk is a consequence of a complex interplay between the host response to infection with a given pathogen, and the invasion and persistence strategy of a second pathogen. While some pathogens do simply co-occur as a result of shared environmental affinities and stochasticity (that is, simultaneous but independent ecological fitting for pathogens invading host species), co-infection can also be a product of interspecific pathogen–pathogen and host–pathogen interactions [3]. These interactions may include direct or indirect processes within the host, such as through toxin-mediated competition [7,8] or immune modulation [9,10], which may alter the likelihood of co-infection depending on the order of infection [11–13]. Alternatively, co-infections may also occur through co-exposure or shared vectors [14]. As such, efforts to characterize pairwise associations between pathogens have risen [3,5]. Beyond pathogen–pathogen interactions, research has also expanded to account for within-host pathogen and microbial community dynamics [15,16], adding additional layers of complexity to understanding the drivers of co-infection.

Acknowledging the patterns and processes that drive co-infections throughout the diversity of host–parasite systems and the potential for interspecific pathogen–pathogen and pathogen–microbiota interactions is paramount, since they can have a cascading, and often detrimental, effect on the course of disease in a population. This can occur through various mechanisms such as by altering pathogen shedding patterns and increasing the risk of transmission [17,18], impairing host immunity and increasing host susceptibility to subsequent illnesses [19,20], or intensifying disease severity [21–23]. With the multifaced ways changes in climate drive pathogen spread and emergence, evaluating how environmental drivers may change the frequencies of co-infections across populations and their geographical distribution is also at the forefront of co-infection research [24–28].

Sound inference for understanding the patterns and processes of when and how co-infections occur from cross-sectional surveillance data requires statistical modelling approaches capable of handling multiple response variables. Fortunately, a wide array of methods is already available for these tasks. Some commonly used methods tailored around classifying infection status as multinomial response variables, for example, enable the comparison of groups by infection status in order to gain insights into variation in the relative frequencies of co-infections versus single infections [27,28]. Another important class of more recently proposed co-infection models include multivariate frameworks that account for conditional dependencies to deal with the joint occurrences of free-living species (‘joint species distribution models’) [29,30]. These have also been adapted to study pathogen community structures [3,31] and microbiome profiles [32,33], as well as to quantify interspecific associations [5,34].

Addressing specific study goals requires careful selection of methods that are well suited to the data and the intended purpose, as the choice will directly impact the ability to draw inferences or make predictions. Unfortunately, determining the suitability of methods is not always straightforward, and this lack of clarity may prevent researchers from making full use of their data’s potential. One additional hinderance that may contribute to this is limited knowledge exchange about conceptual and computational advances between different disciplines, particularly ecology and epidemiology [6,35], which have likely developed largely independently from one another due to differences in host systems and study objectives. To provide clearer guidance for model use in co-infection research, this review seeks to (i) understand the breadth of study goals and host–pathogen systems being pursued with multi-response models and (ii) determine whether there is crossover of knowledge among disciplines. In doing so, we identify challenges and opportunities for expanding the use of these models.

## 2. Material and methods

### 2.1. Data collection

**Figure 1.**
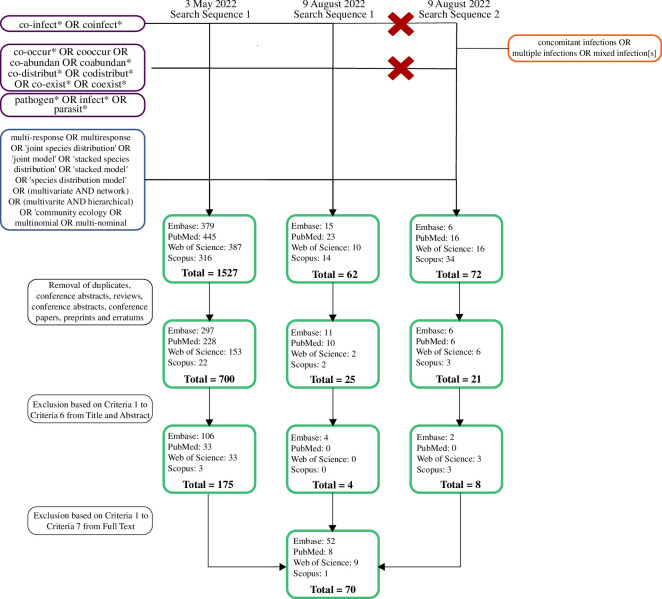
Flowchart of systematic search and filtering process for identifying co-infection studies using multi-response models. Terms to target co-infection studies are presented in purple boxes, and terms to target multi-response models are presented in the blue box. Red crosses indicate terms that were excluded in the second search sequence in order to avoid replicates from the first search when adding the new search terms (in the orange box). To obtain the final paper subset, duplicates were deleted, and papers outside of the scope were filtered first by title and abstracts and then by full texts. A total of 66 papers were kept for data extraction from the first search (3 May 2022), and an additional three papers were added after the second search (9 August 2022).

We conducted a systematic review of papers that used multi-response statistical methods to model co-infection patterns in animal and human populations (see search queries in electronic supplementary material, file 1). Systematic searches were conducted on 3 May and 9 August 2022, across four databases: Embase, PubMed, Web of Science and Scopus (figure 1). Duplicates, as well as papers classified as ‘Conference Abstract’, ‘Conference Papers’, ‘Erratum’, ‘Preprint’ and ‘Review’ in ‘Type of Work’, were removed from the initial search results, leaving 746 out of 1661 papers for screening. Papers were screened using seven criteria, which included studies that (i) were peer-reviewed or primary studies, (ii) utilized observational data, (iii) involved multiple species or variants of infective agents, (iv) involved two or more pathogens, (v) measured infection patterns (i.e. presence–absence or abundance of infections with multiple pathogens of host individuals or populations) as the response variable, (vi) used multi-response methods for analysis and (vii) focused on pathogens of animals or humans. Based on these criteria, 187 papers were retained after screening titles and abstracts only and a final selection of 69 papers after screening the full papers. A full list of these papers can be found in electronic supplementary material, file 3.

### 2.2. Data extraction and validation

Data relating to four study features were extracted from eligible papers: (i) model type, (ii) study goal(s), (iii) purpose, and (iv) study field indexing. Data on ‘model type’ were extracted to summarize the modelling approaches used to analyse co-infection data with multiple outcomes. This study feature encompassed six analytical strategies that were not mutually exclusive, and summarized whether multinomial, multivariate, permutation, classification, network and clustering approaches were used. ‘Study goal’ classified the primary aims of studies for modelling co-infections, grouping studies into ‘association/interaction’, ‘risk factor’, ‘community structure’, and ‘spatial distribution’. The study feature describing the purpose of models was denoted by a single binary variable called ‘prediction’, where a value of 1 was used to indicate whether models were used for prediction in addition to inference, and a value of 0 to indicate inference only. ‘Study field’ classified studies as either ‘Ecology’ or ‘Epidemiology’ based on either journal indexing, study keywords, author affiliations or terminology within the paper. Definitions of variables for model types, study goals and purpose are described in table 1. In addition, a pairwise citation matrix, denoting which pair of studies cited each other, was computed as a proxy for levels of information sharing between included studies. Citation records for each study were manually obtained from the reference list of each study.

**Table 1.** Definitions of different categories of study features used to distinguish studies in terms of model type, study goals and purpose.

variable	definition
**model type**	
multinomial	models where the response variable is categorical and can include two or more categories. One category is used as a baseline reference category. This model assumes that each category is independent of each other and that the outcome is influenced only by the predictors in the model
multivariate	models where multiple response variables are estimated simultaneously, that is, the infection statuses of hosts for different pathogens are considered as separate response variables. These models account for correlations between the outcome variables and seek to understand the effect of predictors on the entire set of outcome variables
permutation	permutation models are methods that use randomization techniques to assess the significance of an observed pattern in the response variable. In the context of co-infections, they are often used to understand whether the observed frequencies of co-occurrences among different groups or across predictor gradients are likely to be due to chance
classification	classification models are models that use a statistical or machine learning algorithm to categorize data into predefined categories. These models use predictors to make decisions about which category an outcome is most likely to belong to
network	network/graphical models are models that are used to characterize the pairwise relationships between objects of interest. In the case of co-infections, the nodes or vertices in an acyclic graph would represent individual pathogen species or variants, and the edges the relationships with each other in terms of co-infection frequencies
clustering	clustering models are statistical models that are used to group data points based on similarities
**study goals**	
association/interaction	association/interaction refers to the goal of measuring and/or quantifying the relative association strength of different pathogens in terms of co-infection frequencies given in the cross-sectional data. Note that, associations refer to the patterns of co-occurrence that are observed, while interactions refer specifically to the effect that one pathogen has on another and vice versa. Papers with either objective have been grouped for the purpose of this study, but the terms are not interchangeable
risk factor	risk factor studies seek to measure and/or quantify the relationships between infection status (e.g. risk of co-infection versus single infection) and predictor such as host or environmental attributes characterizing all samples from the cross-sectional survey
community structure	studies seeking to measure and/or quantify the relationships between pathogen species or variants within a microbial community or understand the (meta)community composition in which a pathogen is situated, either within a single host or across an entire host population
distribution	distribution studies refer to those seeking to understand the geographical patterns of pathogens and co-infections
**purpose**	
prediction	binary variable to indicate whether the purpose of the model was for prediction in addition to inference (1) or inference only (0)
**study field**	
ecology	study field denoted by (i) journal indexing, (ii) paper keywords, (iii) author affiliations, (iv) related text or (v) relevant fields
epidemiology	study field denoted by (i) journal indexing, (ii) paper keywords, (iii) author affiliations, (iv) related text or (v) relevant fields

To verify the accuracy of the data, both the screening phase and the data extraction phase were externally validated by peers on 40 papers from the search results (approx. 5%), and 17 papers from the eligible subset (approx. 25%) of papers, respectively (see electronic supplementary material, file 2).

### 2.3. Identifying natural study clusters and assessing correlation with study discipline

A primary goal of our review was to assess whether co-infection studies tend to be clustered with respect to their study goals and methods used for analysis. To identify natural clusters of studies based on the features, principal component analysis (PCA) was conducted. This clustering analysis was performed using nine binary variables related to model type, study goals and purpose (i.e. indexing focal categories into single binary variables).

In addition to PCA, partitioning around medoids (PAM) was used to divide the studies into two groups, using Jaccard distance (d_J_) as a dissimilarity metric to assess whether clustering resembles study field groupings. This metric was computed from the nine binary variables to measure the dissimilarity between any two given studies (A and B) such that


$$d_{J}\left(A,B\right)=\frac{\left|A\cup B\right|-|A\cap B|}{|A\cup B|}$$

where the intersection (∩) denotes the number of shared attributes and the union (∪) denotes the number of attributes present in either A or B. The Jaccard distances with a value approaching 0 represent studies with shared attributes and those approaching 1 represent studies with no shared attributes. This dissimilarity metric was used in the PAM analysis to identify two clusters of minimal distances. The two clusters generated through this approach were compared to the manual indexing of papers using cross-tabulation and graphical visualization to assess similarities in groupings. All clustering analyses were conducted using the *vegan* [36]*, cluster* [37] and *factoextra* [38] packages in R version 4.3.2.

### 2.4. Quantifying the association between model type and study goals

To better understand which models are favoured among studies with different goals, we quantified the relationships between study goals and model choice, irrespective of cluster assignment. To guide variable selection, pairwise correlations between study goals and other predictors were examined using the *corrplot* package [39] prior to conducting the analysis. The ‘community structure’ and ‘risk factor’ study goals were negatively correlated (−0.68), and therefore the ‘risk factor’ study goal was excluded from the final multivariable model. A positive correlation between ‘prediction’ as the study purpose and the ‘spatial distribution’ study goal was also found (0.63), and so we removed the variable for prediction from the final model. The final adjusted model included three study goal variables (community structure, pathogen associations and spatial distribution), in addition to the variable denoting human (as opposed to animal) hosts. The outcomes of the model were classified into one of three categories: ‘multinomial’, ‘multivariate’ or ‘other models’. One paper that included both multinomial and multivariate models was excluded from the analysis (*n* = 68). The key differences between multinomial and multivariate models are described in Box 1.

Box 1.
**Key differences between multinomial and multivariate modelling frameworks.**

**Figure:**
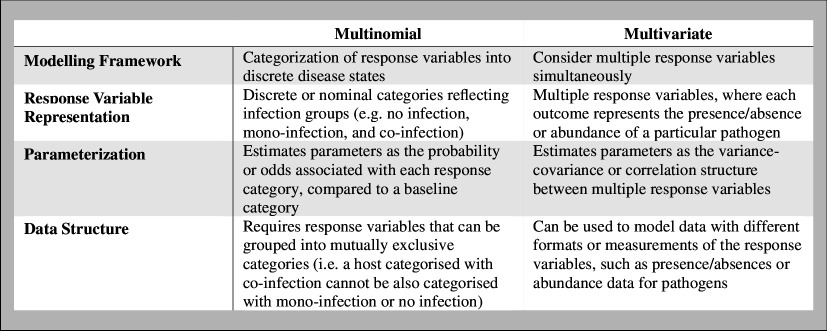

The adjusted Bayesian multinomial logistic regression model was conducted using the *brms* and *cmdstanr* packages in R [40,41]. These models were built using the ‘categorical’ likelihood family. Priors with a normal distribution with a mean of 0 and a standard deviation of 1 were specified for the regression coefficients in the models. Odds ratios were calculated for each variable in all three models by taking the exponential of the coefficient and confidence as 95% highest posterior density confidence intervals. Additional information on variable selection and the model equations can be found in electronic supplementary material, file 4.

### 2.5. Citation analysis

To evaluate the extent of inter-disciplinary collaboration, a directed citation network analysis was conducted using citation record information. The cluster groups determined by PAM as well as the three variables for model type were included as node features in the analysis to explore whether they were linked to network topology. Eigenvector centrality was calculated as an individual-level centrality measure to quantify node importance, and modularity was calculated as a community-level metric to measure the degree of clustering within the network with respect to cluster membership and model membership. This analysis was conducted using the *igraph* [42,43] and *ggplot2* [44] packages in R.

All data and code used in the analysis of this study are openly available in the Zenodo repository (see link in data availability statement).

## 3. Results

A total of 69 papers published between 2005 and 2022 were eligible for the study [3,5,15,25–28,31,34,45–104]. A considerable proportion of studies were published in 2018 or later (39 papers), showcasing that modelling co-infection is an emerging research frontier. The details for each paper and their identification number as referred to throughout the results can be found in electronic supplementary material, file 3.

Most of these studies used cross-sectional data (approx. 88%), quantified pathogen occurrences using binary data (approx. 97%) and studied co-infections of human hosts (approx. 72%). The subset of eligible papers in this review encompassed a broad variety of study goals, host species and modelling approaches. For example, Mair *et al*. sought to identify associations between pathogens in human populations using only multivariate modelling approaches [75]. Aivelo & Norberg similarly sought to describe associations between pathogens as well as between pathogens and other commensal species in animal hosts [15]. In contrast, multinomial models were often used to identify risk factors of co-infections, such as was done in an animal host by Pigeault *et al*., or to predict the spatial distribution of co-infections [87], as was done in human hosts by Soares Magalhães *et al*. [25]. Additionally, some studies explicitly sought to address multiple goals, such as Dallas *et al*., who sought to identify both associations between pathogens and risk factors using multivariate models in multiple animal host species [59]. Others used a combination of methods to address a single main goal, such as Choi *et al.*, who used a combination of network models, permutation methods and clustering to understand the community structure of pathogens in human hosts [57].

Of the 69 co-infection studies, 40 (58.0%) used multinomial models, 17 (24.6%) used multivariate models and 13 (18.8%) used only other models. Of the 19 studies that modelled co-infections in animal hosts, 3 (15.8%) used multinomial models, 10 (52.6%) used multivariate models and 6 (31.6%) used other models. In contrast, in the 50 studies modelling human co-infections, 36 (72.0%) used multinomial models only, 6 (12.0%) used multivariate models only and 7 (14.0%) used other models. One study modelling human co-infections used both multinomial and multivariate models.

### 3.1. Current co-infection research diverges into ecology and epidemiology clusters

**Figure 2.**
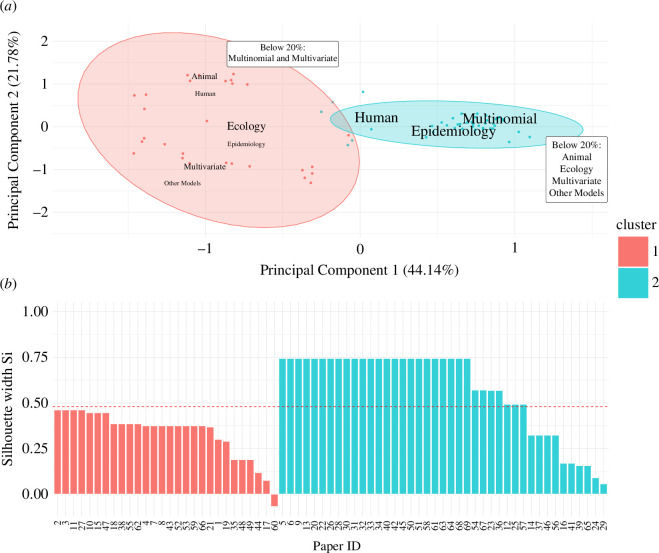
Principal component analysis (PCA) and silhouette plot. This combined plot showcases the cluster groupings obtained through partitioning around medoids (PAM) analysis applied to the dataset of 69 papers. The two clusters are depicted, with cluster 1 in orange and cluster 2 in blue. (*a*) PCA plot. This plot shows the two clusters reduced in a two-dimensional space, whereby each point represents a study according to its PC1 and PC2 values. The cumulative proportion explained by PC1 and PC2 is 65.92% (44.14% by PC1 and 21.78% by PC2). The text within each cluster indicates the host, indexed discipline and model type of studies within each cluster scaled by the proportion. Variables with a proportion below 20% are listed in the textbox beside each cluster. (*b*) Silhouette plot. This plot depicts the silhouette coefficient of papers as obtained through PAM as a measure of the quality of clustering. Each paper is depicted as a vertical bar, with the height determined by the silhouette width, indicating the paper’s similarity to its assigned cluster. Positive silhouette width values approaching 1 indicate well-clustered papers, while negative values approaching −1 suggest that these papers may be better assigned to a different cluster. The average silhouette widths of clusters 1 and 2 are 0.33 and 0.58, indicating fair and good cluster matching respectively, while the total average silhouette width is 0.48, depicted by the dotted red line.

PCA was conducted using variables relating to study goals, prediction and model types to identify natural clusters in the data. Principal components (PCs) 1 and 2 captured the variance in the data relatively well, with a cumulative proportion explained value of 65.92%, showcasing a strong divide in multivariate studies originating mostly from the field of ‘Ecology’ and multinomial studies mostly originating from the field of ‘Epidemiology’ (figure 2*a*). The strongest positive loading factors associated with PC1 with values above 0.2 were for the variables relating to ‘multinomial’ models (0.55) and the ‘risk factor’ study goal (0.47). The variables with the strongest negative factor loadings were the ‘association/interaction’ (−0.39) and ‘community structure’ (−0.39) study goals, along with those associated with the use of ‘multivariate’ models (−0.29) and ‘other models’ (−0.26). For PC2, the variables with the strongest positive loading factors above 0.2 were ‘other models’ (0.47) and the ‘community structure’ study goal (0.39), while the variables with the strongest negative factors were ‘multivariate’ models (−0.56) and the ‘association/interaction’ study goal (−0.46).

The resulting PAM clusters contained 28 (40.6%) and 41 (59.4%) papers, respectively. The average silhouette width between both clusters was 0.48 (0.33 for cluster 1 and 0.58 for cluster 2) (figure 2*b*). Notably, 67.9% of cluster 1 was comprised of ‘Ecology’ papers and 95.1% of cluster 2 was comprised of ‘Epidemiology’ papers according to their study field attributes (figure 2*a*). In cluster 1, 57.1% of studies dealt with animal hosts opposed to 7.3% of studies in cluster 2. In terms of model use, 53.6% of studies in cluster 1 used multivariate models, 3.6% used both multivariate and multinomial models, 42.9% used other models. In contrast, 95.1% of studies in cluster 2 used multinomial models, 2.4% used multivariate models and 2.4% used other models.

### 3.2. Division between study goals and model types used in each discipline

**Figure 3.**
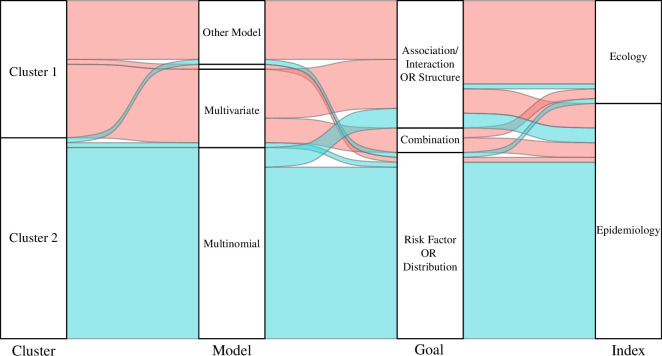
Parallel plot visualizing four study features of the 69 papers included in analysis. Alluvia (bands spanning the plot) are coloured by cluster, with cluster 1 depicted in orange and cluster 2 in blue. The widths of the alluvia correspond to the proportion of papers that belong to the strata on the right side of the band, given their placement on the axis to its left. The left axis represents the cluster assigned through partitioning around medoids (PAM), and contains two strata for cluster 1 and cluster 2. The middle-left axis represents model type, stratified into four categories: ‘other models’, ‘multinomial & multivariate’ (not labelled on plot due to size), ‘multivariate and ‘multinomial’. The middle-right axis represents study goals, grouped and stratified into three categories: (i) those where ‘association/interaction’ and/or ‘community structure’ were the only study goals, (ii) those where ‘risk factor’ and/or ‘spatial distribution’ were the only study goals, and (iii) those that combined goals from (i) and (ii). The right axis represents the manual indexing of papers into disciplines, with two strata for Ecology and Epidemiology.

With regard to model type, all papers using multinomial models only were assigned to cluster 2 (i.e. the ‘Epidemiology’ cluster), while 12 of the 13 papers (92.3%) that used other models were assigned to cluster 1 (i.e. the ‘Ecology’ cluster) (figure 3). Of the 16 papers that used multivariate models only, 15 (93.8%) were assigned to cluster 1. The one study that used multivariate models that were assigned to cluster 2 looked at risk factors or spatial distribution as a study goal. Of 26 papers that looked at either pathogen associations or community structure only, 22 (84.6%) were grouped into cluster 1. Thirty-seven of the 38 papers (97.4%) that looked either at risk factor identification or spatial distributions were assigned to cluster 2. All five studies combined both pathogen association or community structure goals with risk factor identification or spatial distribution analysis assigned to cluster 1. Only one paper used both multinomial and multivariate models, which was assigned to cluster 1 but was indexed as ‘Epidemiology’. Of the remaining 47 papers indexed as ‘Epidemiology’, 39 (83.0%) were assigned to cluster 2. Of the 21 papers indexed as ecology, 19 papers were assigned to cluster 1 (90.5%). Of the two that were assigned to cluster 2, one used multinomial models to study risk factors or spatial distributions, and the other used multinomial models to understand associations or community structure.

Independent from the discipline clusters, the results from the Bayesian multinomial logistic regression model further suggest that the study goals are associated with model choice between the three model types (table 2). Specifically, studies that sought to detect pathogen associations were more likely to use multivariate models over multinomial models (OR = 17.04 [5.42, 56.87]). Studies that sought to understand pathogen or pathogen–microbial community structure were more likely to use other models compared to multinomial models (11.74 [3.52, 41.66]). No associations were found between host type and the spatial distribution study goal in the final adjusted model. Additionally, studies that involved human hosts were less likely to use multivariate models over multinomial models (OR = 0.26 [0.07, 0.95]).

**Table 2.** The relative use of multinomial versus multivariate or other models for co-infection studies for different goals. Given as the estimates of odds ratios from Bayesian multinomial logistic regression model using study goals to predict model type with multinomial models as the baseline (*n* = 39). Estimates in bold represent 95% confidence intervals that do not include 0.

	multivariate (*n* = 16)		other models (*n* = 13)	
	OR estimate	95% CI	OR estimate	95% CI
**study goals**				
human host	**0.26**	**0.07, 0.95**	0.69	0.18, 2.62
associations/interaction	**17.04**	**5.42, 56.87**	1.44	0.40, 5.09
community structure	1.24	0.32, 4.65	**11.74**	**3.52, 41.66**
spatial distribution	1.12	0.27, 4.58	0.5	0.10, 2.33

### 3.3. Limited knowledge exchange between disciplines

**Figure 4.**
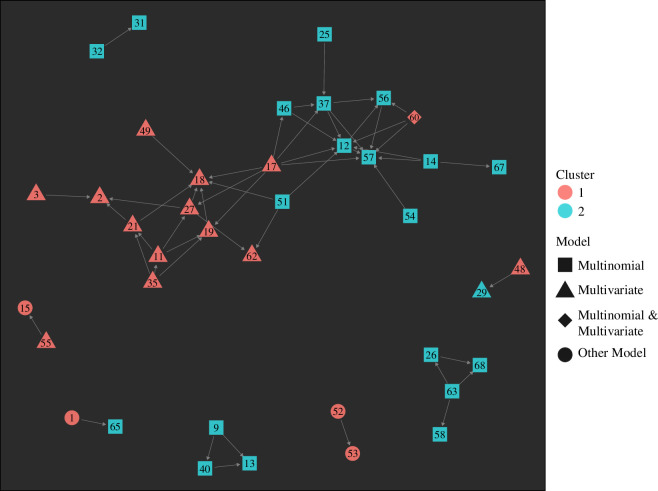
Citation network showing citations between those 39 out of the 69 papers connected by other studies by citations (30 papers excluded from graph due to no citations of other papers within the subset of papers for analysis). Nodes are characterized by model type, where squares represent multinomial models, triangles represent multivariate models, diamonds represent multinomial and multivariate models and circles represent other models. Numbers within nodes correspond to the paper ID numbers listed in electronic supplementary material, file 3. The colour of nodes corresponds to model type where orange nodes represent papers assigned to cluster 1, and blue represents papers assigned cluster 2 in the PAM analysis. The main network is comprised of 22 nodes, 12 from cluster 1 and 10 from cluster 2.

Of the 69 papers in this review, 39 papers (56.5%) cited, or were cited by, at least one other paper in the eligible subset, which are shown in figure 4. Brooker *et al*. [27], Raso *et al*. [93], Clark *et al*. [5] and Soares Magalhães *et al*. [25] were the most cited or citing papers of others within this citation network and were thus most central to the citation network, as indicated by eigenvector centrality values of 1.00, 0.89, 0.87 and 0.82, respectively.

Within the citation network, 18 papers belonged to cluster 1, accounting for 64.3% of papers within the cluster, while the remaining 21 papers accounted for 51.2% of cluster 2. Of those papers that used multinomial methods, 21 papers (52.5%) were connected in the citation network, whereas 15 papers (88.2%) that used multivariate models, and four papers (30.8%) that only used other methods were connected in the citation network.

To evaluate network division based on cluster membership and model use, two modularity values were calculated for the subnetwork represented by the 39 papers. Moderate network clustering was found based on cluster membership (i.e. papers belonging to either cluster 1 or 2) and model type use (i.e. multinomial, multivariate, multinomial and multivariate or other models) with modularity values of 0.30 and 0.35, respectively.

## 4. Discussion

While a multitude of models exists for modelling patterns in multiple response data, their applications in co-infection research for understanding different study goals and approaches used across disciplines have been unclear up to this point. This systematic review does not only suggest a strong link between specific study goals and the choices between the most commonly used model types, but also highlights a clear divergence in research focus among ecologists and epidemiologists, calling for better cross-disciplinary approaches in future research endeavours. Specifically, we found that the majority of studies that sought to understand the role of risk factors on co-infection, and those that sought to model the spatial distribution of co-infections were predominately linked to multinomial models being the most often used in studies from the field of epidemiology, whereas studies that sought to understand individual associations or interactions between pathogens were found to be linked to multivariate model use, and those that sought to understand the overall structure or composition of pathogen communities were linked to the use of other multi-response models, as shown in table 2. These findings reinforce the notion that specific and distinct goals and methods are studied between the two disciplines.

The results from the citation analysis further suggest that cross-disciplinary collaboration and exchange among epidemiologists and ecologists is currently limited, as summarized in Box 2.

Box 2.
**Summary of modelling frameworks including their current use in ecology and epidemiology, and opportunities for cross-disciplinary collaboration.**

**Figure:**
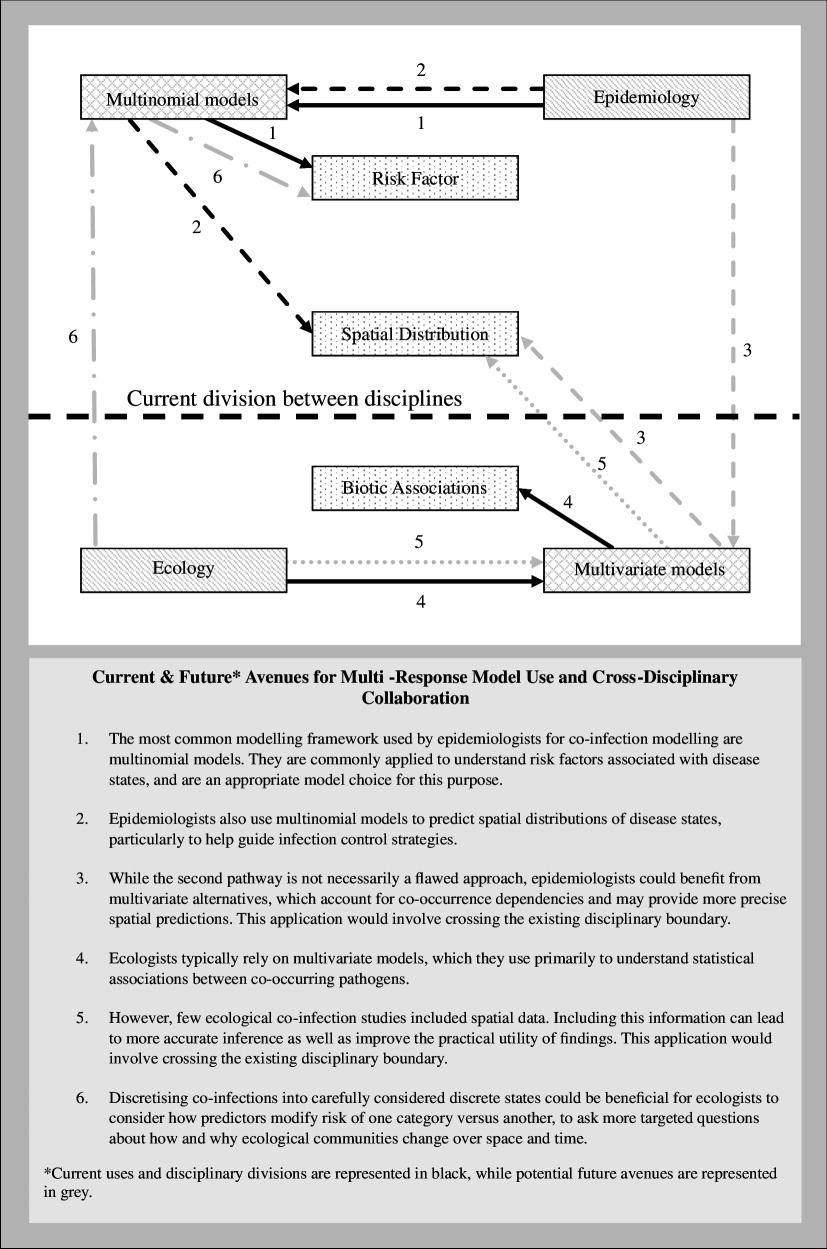

­

These findings corroborate previous calls for increased collaboration between the two disciplines, which have highlighted the limited overlap between work by epidemiologists and ecologists working on parasite communities in human and animal hosts [6] and multi-species infection dynamics [35]. Similar findings have previously been documented in other fields, with one study conducting a similar citation network noted the limited citating practices of researchers of work outside of the discipline [105], suggesting that the siloing of information within disciplines might be one barrier to cross-disciplinary collaboration. Moreover, the findings from our citation network also highlighted limited citations between studies that used multinomial models and multivariate models. These findings could suggest that a lack of awareness of different model types may drive study objectives, rather than model choice being driven by the study goals, marking a unique opportunity for the sharing of knowledge on these methodologies. This is consistent with the growing recognition of interdisciplinary research and transdisciplinary collaboration for better understanding and control of infectious diseases, with increasing momentum growing for collaborative approaches, such as the One Health framework, which recognizes the interconnected nature between the health of humans, animals and the environment [106]. By increasing the sharing of modelling practices between in ecology and epidemiology, researchers have the potential to instigate more profound discussions on their practical application, embracing a more holistic framework to contribute to a more resilient and integrated approach for disease management.

A clear preference among epidemiologists for multi-response modelling of co-infections, multinomial models are an effective tool for capturing risk factors associated with individual-level risk. As highlighted in this review, this was the most common study goal in studies indexed as epidemiological and is a more suitable choice over multi-response modelling approaches, such as multivariate models, for this task, because they enable easy quantification of the difference in disease risk attributable to specific exposures with commonly used statistical models. This is a particularly useful approach for evaluating behavioural risk factors associated with particular types of infections, such as sexually transmitted infections (STIs), such as, for example, Culbreth *et al*. who explored how engaging in drinking and sexual behaviours is associated with self-reported HIV–STI co-infection among youths [58], and Fotiou *et al*. who evaluated the association between drug injecting behaviours and HCV–HIV co-infection [64]. Alternatively, a similar modelling approach was used to describe the clinical profiles associated with co-infection in de Souza *et al*., who applied these methods for the purpose of associating symptoms for classifying viral and bacterial respiratory co-infections [60]. Because of the interpretability that these models offer, they also allow for comparison between multiple models as well, such as Binka *et al*. who built multinomial models for different ethnic groups to compare the difference in risks associated with co-infection with hepatitis B, hepatitis C and HIV co-infection [51].

Besides risk factor identification, another study goal commonly pursued by epidemiologists included modelling the spatial distributions of co-infections. Among these studies, multinomial models were also the preferred method for understanding the geographical spread of disease. This was especially popular among researchers looking at helminth co-infections in human hosts, making up nine of the 12 studies that sought to model spatial distributions using multinomial models [25–27,55,63,90,92,93,102]. These methods are effective at capturing the co-infection patterns and detecting locations associated with no infections, mono-infections and co-infections, allowing researchers to draw inferences from the data and identify hotspots areas. Of these nine studies, seven also sought to predict areas associated with higher infection risk at the population level using these models [25–27,55,63,92,93]. Only two of the 12 studies applied multivariate models to inform predictions [5,96]. Interestingly, one of these studies used both a multinomial model and two-component models: an independent component model that considered probability of single-infection status, and a shared component model, which considers conditional dependencies between diseases through location-specific shared components [96]. While the study found the multinomial model to outperform the other models across most performance metrics, it was not able to be applied to single-disease survey data. In these cases, the shared component model showed the best performance over the individual models. This, therefore, represents an underexplored opportunity for epidemiologists to leverage existing data from single-disease surveys to formulate better predictions on disease risk by accounting for potential conditional dependencies between multiple pathogen occurrences.

Clark *et al*., who utilized a multivariate model for predicting spatial distributions, also demonstrate the utility of a multivariate network model called conditional random fields, revealing a comparable performance for predictions of human helminth infections at the individual level between the model and single-parasite gradient boosted machine models, and higher predictive accuracy at the school level [5]. These findings highlight the potential of using these models as a way of accounting for conditional dependencies within the models to generate accurate predictions. While inconsistencies in predictive performances of different multivariate models have been noted [107–116], these models have been shown to often lead to more accurate predictions of the co-occurrence of free-living species and plants, particularly for rare species [110,111,115,116]. These models are therefore also worth considering for modelling the geographical distributions of co-infections, either in place of multinomial models for predicting high-risk areas, or in addition to multinomial models when also seeking to make inferences about risk factors. Moreover, these methods provide a useful avenue for utilizing big data with large numbers of pathogens (and microbiomes) that might be oversimplified if grouped into a few broader categories, as would be feasible for multinomial modelling.

Accounting for conditional dependencies between pathogens using multivariate modelling was included more frequently in studies indexed as ecological; however, these models were primarily used to measure pathogen–pathogen associations as opposed to predicting spatial distributions of co-infections. One popular method for doing this was the hierarchical modelling of species communities (HMSC) framework [15,46,53,59,73], a joint species distribution model (JSDM) that accounts for interspecific associations within a residual variance–covariance matrix [117]. While this method has been previously applied to incorporate spatial data for free-ranging organisms [118,119], our review highlights that these models have primarily been used to quantify interactions between pathogens (i.e. the co-occurrence of pathogens in host individuals, regardless of the spatial context of the infections). Only one study accounted for conditional dependencies by including information about the spatial context of sampled host individuals [46], while no study applied this model to predict the geographical distributions of pathogen co-occurrences. Expanding the study goals and including spatial parameters within these models offer ecologists the opportunity to improve the utility of the models for practical applications and create an overlap with the research interests of epidemiologists, that may subsequently help improve collaboration practices between the two disciplines.

In relation to the fourth study goal identified in this review, understanding pathogen community composition, the findings suggest that other methods, including permutation analyses, network analyses, clustering and classification methods are preferred by researchers over multinomial or multivariate models. Notably, of the 18 studies that sought to understand pathogen community structure, nine studies also included commensal species in addition to pathogenic species [15,46,52,57,78,80,83,88,101]. One such example is by Bouillaguet *et al*., who sought to characterize the microbiota associated with apical periodontitis using a combination of network, permutation and clustering methods [52]. Given that the inclusion of these commensals introduces an additional layer of complexity to understanding co-infections, the choice of methods in these instances may be reflective of the number of species (i.e. response variables) included in the analyses. However, while we have included these studies within the scope of this review to showcase the breadth of co-infection research that is being pursued, further investigation into this subset of studies would be required to provide guidance on model suitability for the task and discipline-specific recommendations. Moreover, while the binary indexing categories for disciplines as ‘Ecology’ and ‘Epidemiology’ were utilized here to describe two common fields concerned with infectious diseases for the purpose of this review, allowing generalizability of the findings within these two disciplines, these classifications may be overly simplistic to draw accurate conclusions regarding this subset of studies looking at community structure and may be better represented under another classification such as ‘Microbiology’.

While the findings from this study have the potential to serve as a guide for the use of multi-response models for co-infection research and future avenues for cross-disciplinary collaboration, the limitations of this study should also be noted. First, this study focuses on the use of statistical models for modelling observational co-infection data and does not consider mechanistic models within its scope. With regards to the analyses conducted in the review, it should be noted that the odds ratio values from the Bayesian multinomial logistic regression models are not reliable quantitative measures but are deemed fit for purpose here to showcase the divergence in model applications. In terms of the citation network, it should also be noted that there is bias regarding the number of citations pertaining to each study, and in particular studies containing no citations, given that more recent publications have had less time to be cited and therefore the number of citations is likely to be correlated to the year of publication. Thus, cross-collaboration between disciplines within the network cannot be inferred for more recent studies from the citation network alone. Nevertheless, the citation analysis included in this study provides a good indication of trends occurring with earlier work. Lastly, while our systematic approach aimed at providing a comprehensive overview of relevant literature, the degree of variability in terminology for various modelling approaches means our search is unlikely to be exhaustive, particularly where methods did not include multinomial and multivariate models.

## 5. Conclusion

Selecting statistical models for data analysis can be an arduous task and requires an understanding of how core research questions can be supported by suitable models fit for purpose. The strong divide in both study goals and statistical model choice in studies dealing with co-infections from epidemiological and ecological perspectives highlights that improving communication and sharing of ideas across disciplines is needed if we aim to understand different model systems to an equal extent and aim to synthesize ecological and epidemiological insights together into One Health solutions.

## Data Availability

Data and supporting code to replicate analysis have been made available from the Zenodo repository [120]. Supplementary material is available online [121].
